# Designing a Clinical Study With Dietary Supplements: It's All in the Details

**DOI:** 10.3389/fnut.2021.779486

**Published:** 2022-01-18

**Authors:** Z. Elizabeth Floyd, David M. Ribnicky, Ilya Raskin, Daniel S. Hsia, Jennifer C. Rood, Bill J. Gurley

**Affiliations:** ^1^Pennington Biomedical Research Center, Louisiana State University System, Baton Rouge, LA, United States; ^2^Department of Plant Biology, Rutgers University, New Brunswick, NJ, United States; ^3^National Center for Natural Products Research, University of Mississippi, University, MS, United States

**Keywords:** botanical, natural products, dietary supplements, *Artemisia dracunculus*, clinical trial

## Abstract

A successful randomized clinical trial of the effect of dietary supplements on a chosen endpoint begins with developing supporting data in preclinical studies while paying attention to easily overlooked details when planning the related clinical trial. In this perspective, we draw on our experience studying the effect of an ethanolic extract from *Artemisia dracunculus* L. (termed PMI-5011) on glucose homeostasis as a potential therapeutic option in providing resilience to metabolic syndrome (MetS). Decisions on experimental design related to issues ranging from choice of mouse model to dosing levels and route of administration in the preclinical studies will be discussed in terms of translation to the eventual human studies. The more complex considerations in planning the clinical studies present different challenges as these studies progress from testing the safety of the dietary supplement to assessing the effect of the dietary supplement on a predetermined clinical outcome. From the vantage point of hindsight, we will outline potential pitfalls when translating preclinical studies to clinical studies and point out details to address when designing clinical studies of dietary supplements.

## Introduction

Over one-half of all adults in the United States report using a dietary supplement to improve their health (1). This trend coincides with increasing interest in the health benefits of plant-based natural products and a burgeoning number of preclinical studies investigating the therapeutic properties of extracts derived from botanical sources. While animal model-based pre-clinical studies are expected before moving to clinical studies, there is ample data showing these pre-clinical models often do not translate in terms of efficacy or safety to human studies (2). Our view is that reliable information about the safety and efficacy of botanically-based dietary supplements in humans requires clinical studies that build on well-designed preclinical results that are relevant to the targeted human population. To accomplish this goal, the eventual clinical trial should be kept in mind when designing the preclinical study. Our experience studying the metabolic effect of an ethanol extract from *Artemisia dracunculus* L. (termed PMI-5011) points out the myriad details that have a substantial impact on the ability to translate preclinical findings to the clinical setting and the additional details that must be considered when designing the related clinical study. In this perspective, we offer some hard-won advice based on our stumbles and successes with investigating how dietary supplementation with PMI-5011 impacts obesity-induced insulin resistance.

## Designing the Preclinical Study: Think Ahead To The Clinical Trial

The ability to translate preclinical studies of dietary supplementation with botanical extracts fundamentally begins with quality control of the plant material. Plants grown for preclinical studies of botanical extracts should be cultivated under uniform conditions in a controlled growth environment to yield consistent plant material over time. The growth conditions should be pesticide-free as a safety consideration for eventual human consumption (3). Bioactivity-guided fractionation (4) or DESIGNER (Deplete and Enrich Select Ingredients to Generate Normalized Extract Resources) (5) approaches can be used to define the active fraction and provide a biomarker for evaluating each batch of the plant material to ensure quality control. Our experience is that batch-to-batch variation in the selected biomarker will occur even with quality control practices in place and may be traced back to seed lot, disruptions in the growth conditions, storage conditions, or variability in the extraction method. In addition, the stability of identified bioactives may change over time with fractionation of the complex mixture of phytochemicals when compared to the parent extract. In each case, having a reliable biomarker of the biological endpoint of interest is essential for extract content analysis, interpreting experimental results, and carrying out pharmacokinetic studies in animal models or clinical trials.

Our research with *Artemisia dracunculus* L. provides an example of identifying an extract-derived biomarker that is strongly associated with the biological endpoint of interest. Our studies focus on the ability of botanical extracts to impart resilience to developing risk factors for type 2 diabetes associated with obesity. The metabolic syndrome (MetS) is a prediabetic state characterized by insulin resistance and elevated blood glucose along with elevated triglycerides, reduced high-density lipoprotein (HDL) cholesterol, elevated blood pressure, and abdominal obesity (6). Clinically, MetS is defined by the presence of at least three of the five risk factors. This illustrates the need to determine the important endpoints that are feasible to assay in preclinical studies and are clinically translatable. In our example, a focus on insulin responsiveness during screening and the subsequent activity-guided fractionation experiments offered the best opportunity to capture a preclinical effect (e.g., signaling responses to insulin in skeletal muscle cells *in vitro* and *in vivo*; blood glucose and insulin levels *in vivo*) that is commonly measured in the clinical setting (blood glucose and insulin levels) to evaluate the risk of developing type 2 diabetes. Using these endpoints, bioactivity-guided fractionation of an ethanolic extract of plant shoots obtained from *A. dracunculus* during the flowering stage (termed PMI-5011) identified a fraction containing four potential bioactive compounds (4, 5, 7–9). 4-*O*-methyldavidigenin was subsequently identified as a reliable extract biomarker for the biological activity of interest *in vitro*, including cell culture of human skeletal muscle cells, which reflect *in vivo* biology (10–13).

Moving from cell-based studies to animal models brings another layer of issues to consider. Choice of an animal model has to be made with the human target population in mind. Our interest in obesity-related insulin resistance and type 2 diabetes prompted us to use the KK-A^y^ genetic model of hyperglycemia in the initial *in vivo* experiments (9, 14). Although the KK-A^y^ mouse is a robust model to test our supplement, a genetic model of hyperglycemia does not capture the early phases of obesity-related changes in insulin sensitivity that predict developing type 2 diabetes. To better align with the clinical picture of MetS, later preclinical experiments were carried out in a rodent model of obesity-induced insulin resistance (15, 16). However, the insulin resistance phenotype in the C57BL/6 mouse model of obesity-induced insulin resistance occurs in obese male, but not female mice when fed a defined high fat diet (17).

Subsequent studies using both male and female mice show striking sex-dependent differences in response to dietary supplementation (17, 18). Given the evidence that dietary supplements are often consumed by women across the age and health spectrum (1), this points out the importance of using both sexes in pre-clinical rodent studies unless the target population is only men or women. Experimental variability is not greater in fertile female mice compared to male mice (19). This alleviates much of the concern about including female mice in pre-clinical studies, but our experience indicates the two sexes should be studied initially as independent cohorts. Inclusion of female mice also highlights the importance of determining the age of the target population. Metabolic syndrome is becoming more prevalent in younger women due to increasing obesity or changing dietary patterns (20, 21). With that in mind, our studies are carried out in fertile female mice rather than simulating the onset of MetS by ovariectomizing young mice or aging a colony of female mice for up to 2 years. An excellent guide for designing pre-clinical studies of sex differences is provided by Mauvais-Jarvis et al. (22).

Formulation of the botanical dietary supplement will also be an issue. Oral administration is a given, but capsules or pills cannot be given to a mouse, so the supplement is typically formulated in the diet. This points out the importance of establishing bioavailability in the pre-clinical animal model. While dietary supplementation with a botanical extract can be incorporated into a rodent diet at a wide range of concentrations, this will not be feasible in clinical trials with humans. Even if the supplement is ultimately administered to humans in gel or capsule form, low bioavailability in the pre-clinical phase may signal that an excessive number of capsules will be required in the clinical trial. Thus, the pre-clinical studies offer an excellent opportunity to optimize bioavailability based on the identified biomarker. When determining bioavailability in rodents, keep in mind the food intake patterns of nocturnal animals. If bioavailability in a fasted state is important, food should be withdrawn for 4–6 h prior to the test. The time of day should also be noted to account for any circadian effects on bioavailability (23, 24).

Diet composition is another important variable. We typically use a defined 45% kcal high fat diet that contains 17% kcal sucrose (Research Diets) as the standard diet for studies of obesity-related metabolic disease. This has the advantage of increased bioavailability of the lipid-soluble bioactives, but more recent studies use high fat (45% kcal), high sucrose (30% kcal) diets that more closely mirror the typical Western diet associated MetS (18). While our interest in obesity-induced insulin resistance requires following the extract's effect as obesity develops, the extract may be given in preclinical studies over a short time to determine acute effects or to carry out pharmacokinetic studies. In our studies, we observe that botanical extract efficacy varies with the length of time administered (unpublished observation). An effect present after 2 months consuming the dietary supplement may not be present after 3 months or longer consuming the same diet and dietary supplement even if the supplement composition is unchanged over time. The pre-clinical stage allows for evaluation of whether the dietary supplement should be taken chronically or will be more effective when taken over shorter periods. If the dietary supplement is given for a short period, it is tempting to administer the supplement using gavage. However, this is stressful for the mouse and adaptation to gavage should be carried out using vehicle only prior to initiating supplementation.

An essential part of pre-clinical studies is evaluating the safety of the chosen botanical dietary supplement. This should include measuring body weight, food intake, activity level, tissue weights, mortality rate, liver morphology at necropsy, and clinical evaluation of hepatic function using albumin, bilirubin, alanine aminotransferase (ALT), and aspartate aminotransferase (AST) serum levels. These findings can be correlated with the bioavailability of the botanical extract. Depending on the disease model of interest, more specific safety questions should also be addressed. Our experience indicating PMI-5011 supplementation with a 45% high fat diet may have adverse effects in female, but not male mice (17) further highlights the need to consider supplement safety in both sexes of the chosen pre-clinical model.

## The Clinical Study: The Nitty-Gritty Details

Clinical trials assessing the impact of nutrition on human health face unique challenges when designing, interpreting, and reporting the study results (25–29). The complexity of botanical extracts increases the challenge of designing clinical studies of dietary supplements, but the ability to build on strong pre-clinical data increases the likelihood of an outcome that provides reliable clinical data. Thus, the first step when considering a clinical trial of the dietary supplement is to ask if there is good evidence from the preclinical studies for moving forward with a clinical study. To answer this question there should be clear evidence of preclinical safety as well as a convincing demonstration of efficacy before transitioning into the clinic. While there are often significant species differences in physiology and pharmacology, any concerns regarding pre-clinical acute and/or chronic toxicity will preclude moving forward to first-time-dosing in humans. Despite a promising ethnopharmacological track record as either a food or natural medicine, pre-clinical botanical safety studies conducted with a well-characterized extract are necessary to allay any concerns when the extract is formulated and administered as a dietary supplement to human subjects.

### Start With Safety, Tolerability, and Bioavailability

Central to assessing the safety and efficacy of a botanical dietary supplement in humans is determining the bioavailability of marker compounds that appear key to pre-clinical effectiveness. Unfortunately, for many phytochemicals, poor oral bioavailability is the rule, not the exception (30–33). This stems from two major factors: human physiology and phytochemical physico-chemistry. From an evolutionary perspective, the ingestion of plants has significantly impacted human development, such that, as a species, we readily biotransform phytochemicals, either through enzymatic metabolism in the gut or liver parenchyma, or via gut microflora (30). Many phytochemicals are also substrates for various efflux transporters expressed on the apical surface of intestinal enterocytes, which can preclude their uptake from the gut lumen. Collectively, actions of these enzymes and transporters reduce phytochemical exposure. On the other hand, many phytochemicals are lipophilic, having insufficient water solubility to become bioaccessible. Combined, extensive pre-systemic metabolism and poor bioaccessibility (efflux transport, lipophilicity) render many seemingly “active” phytochemicals inadequately bioavailable.

### Formulation of the Product

Most botanical dietary supplements on today's market are gelatin or cellulosic capsules filled with dried extract. Whether the extract was derived using an aqueous or non-aqueous procedure can have a significant impact on the “performance” of the dosage form. Non-aqueous extracts (e.g., hexane, ethylacetate, etc.) will recover more highly lipophilic phytochemicals, while aqueous or ethanolic extracts will recover more hydrophilic components. Lipophilic marker compounds will oftentimes have poorer bioavailability due to inadequate solubility in gastrointestinal fluid, especially if taken on an empty stomach. An assessment of dietary supplement dosage form performance is critical before conducting a clinical study, as poor performance will undoubtedly lead to questionable clinical outcomes. Dosage form performance assessment can be accomplished by conducting a disintegration/dissolution study. An overview of dissolution/disintegration guidelines and recommended equipment can be found in the United States Pharmacopeia (34).

Briefly, dosage form performance investigates whether a tablet, capsule or liquid gel capsule can disintegrate and release its contents into gastrointestinal fluids in a timely manner. Dosage forms that quickly disintegrate and readily release their active components generally exhibit better bioavailability than those with inferior performance traits. In effect, this process is an *in vitro* means of gauging the rate and extent of phytochemical release into simulated gastric or intestinal fluids using standardized conditions and equipment (35). Even capsule composition (i.e., gelatin vs. cellulosic) can impact dosage form performance. Old or outdated gelatin capsules may bleb and not adequately disintegrate, whereas certain phytochemicals may induce cross-linking of polysaccharide chains in hydroxypropylmethylcellulose capsules which can compromise disintegration and dissolution (36).

It may also be prudent to incorporate biorelevant media into dissolution studies (37, 38). Biorelevant media are designed to simulate fasted and fed states, two conditions that better emulate the gastrointestinal environment phytochemicals may experience *in vivo*. For lipophilic phytochemicals, dissolution media mimicking fed conditions facilitate micelle formation and enhance solubility, whereas those simulating fasted conditions may yield less favorable results. For other phytochemicals, the presence of food may impair absorption. Incorporation of biorelevant media, therefore, can aid in clinical study design; that is, provide guidance as to whether the supplement should be taken with or without food. The simple task of taking the supplement formulation with food, especially a fatty meal, may preclude the need for developing a novel dosage form specifically designed to improve phytochemical bioavailability (e.g., phytosome, nanoemulsion, etc.) (31). If suitable bioavailability was achieved in pre-clinical studies when the extract was incorporated into the diet, then meals served in the clinical setting should try to reasonably match the carbohydrate, fat, and protein percentages within the animal chow. A word of caution, however, regarding vegetables in meals, for clinical studies is that the potential for phytochemical–phytochemical interactions always exists between the supplement and any plant-based food (39).

### Choosing the Study Population

When selecting participants for a supplement-based clinical study, it is ideal to recruit those representative of the product's intended target population. If the product is designed for women, athletes, elderly, etc., then individuals representing those groups should be targeted; otherwise, healthy volunteers of both sexes covering a range of ages are typically utilized. Exclusion criteria generally include acute and chronic disease, prescription medications (an exception is sometimes made for oral contraceptives, unless there is evidence the metabolism of select marker compounds may be affected), and dietary supplement use.

### Dosing and Timing of Administration

Doses for first-time administration to humans of a botanical extract not currently on the market (i.e., PMI-5011) can be extrapolated from those evaluated in pre-clinical animal studies using allometric scaling. Allometric scaling incorporates appropriate power functions that correlate body surface area and/or weight to various physiological parameters across animal species in order to estimate human equivalent doses (HED). While there is much debate within the scientific literature regarding the best exponent for use in exponential allometry calculations, U.S. Food and Drug Administration (FDA) recommends the following approach: HED = animal NOAEL × (W_animal_/W_human_)^(1−b)^, where NOAEL is the “no observed adverse effect level” for the pre-clinical, animal dose scaling study, W is weight in kg, and b is the allometric exponent equal to 0.67. Utilizing this approach, Table 1 is useful in converting animal doses to HED.

**Table 1:** Converting preclinical supplement doses to human equivalent doses (HED).

**Species**	**To convert animal dose in mg/kg to HED in mg/kg, either:**	
	**Divide animal dose by:**	**Multiply animal dose by:**
Mouse	12.3	0.08
Hamster	7.4	0.13
Rat	6.2	0.16
Guinea pig	4.6	0.22
Rabbit	3.1	0.32
Dog	1.8	0.54
Rhesus monkey	3.1	0.32
Mini-pig	1.1	0.95

As an example, the HED for a 50 mg/kg extract dose (NOAEL) in a 0.02 kg mouse would be 50 ×0.08 = 4 mg/kg, or 280 mg in a 70 kg adult human. It should be noted that these FDA guidelines were developed to determine the maximum recommended starting dose (MRSD) of experimental drugs in humans. It must be emphasized that an HED is simply a starting point, it may or may not need to be adjusted depending upon the drug/phytochemical and its specific biotransformation pathways. Incorporating additional safety factors, however, are often recommended prior to administering an HED derived from animal NOAEL data. Further reducing an MRSD by a factor of 10 or even 100 may be prudent for first-time-use in a clinical safety study. Additional discussions of dose conversion methods for botanical extracts based upon allometric scaling and safety factors can be found in Wojcikowski and Gobe (40) and Schilter et al. (41).

### Planning a Pharmacokinetic (PK) Study

The first-time-in-human dose for a botanical extract should be aimed at determining PK parameters for one or more biologically relevant marker phytochemicals. These parameters include “area under the concentration-time curve” (AUC_0−∞_), maximum blood/plasma concentration (*C*_max_), time to reach *C*_max_ (*T*_max_), elimination half-life (*t*_1/2_), and if possible, apparent clearance (CL_app_). To generate these parameters, it is important to collect a sufficient number of blood levels over a defined period to best characterize the AUC and *t*_1/2_. These time points often mirror those used for the animal study, although subject inconvenience may preclude certain timepoints (i.e., 18 h post-dose). Blood collection times of 0.0, 0.5, 1, 2, 4, 6, 8, 12, 24, and 48 h are fairly common, although these can be modified for subject convenience. To be as efficient and practical as possible, a single dose of extract is administered in the morning (with or without food) and blood sampling will span at least 12 h the first day, with subsequent blood draws at 24 and perhaps 48 h. Given that most phytochemicals have fairly short elimination half-lives in humans, samples obtained after 24 h may not be necessary (30, 32). A clearly defined biomarker as determined by pre-clinical studies is essential for the clinical PK study. Once there is evidence that the marker compound(s) is bioavailable and a half-life can be determined, a multi-dose administration scheme can be devised to assess PK parameters at “steady state.” Marker compound half-lives <6 h will mean that the extract should be dosed at least 3–4 times daily for at least 3–5 days to reach steady state. Longer half-lives will require less frequent dosing but a longer period of administration to achieve steady state.

### Dietary Influences

Diet and concomitant drug use may affect phytochemical PK. Prescription drug and botanical dietary supplement (e.g., St. John's wort, goldenseal, licorice, multi-ingredient supplements, etc.) use are often exclusion criteria, while non-prescription drug use is strongly discouraged. Dietary factors (i.e., vegan diet) and certain dietary restrictions should also be considered, as certain fruits and vegetables can modulate exogenously administered phytochemical metabolism. Cruciferous vegetables (e.g., broccoli, brussel sprouts, asparagus, water cress, etc.) and certain citrus fruits and fruit juices (i.e., grapefruit, pomelo, orange) should be avoided (42). Food diaries are highly recommended for use in multi-dose PK or efficacy studies so that any unanticipated dietary influences can be accounted for (42).

Given that many phytochemicals undergo extensive pre-systemic metabolism, especially glucuronidation, it is plausible that phytochemical metabolites may contribute to a botanical extract's efficacy. In fact, active glucuronide metabolites have been identified for many phytochemicals (43–45). While parent phytochemicals may be below detection limits for some analytical assays, their glucuronides—or indirect evidence of glucuronides when samples are treated with β-glucuronidase—may be quantifiable in plasma or urine. Thus, urine collection should also be considered when conducting both phytochemical marker PK and efficacy studies. Urine collection intervals of 0–4, 4–8, 8–12, and 12–24 h are frequently used to characterize the contribution of renal elimination to phytochemical PK and for active metabolite identification and quantification.

### Regulatory Considerations

From a regulatory perspective, one must consider the study's ultimate goal. An Investigational New Drug (IND) application may need to be filed with the U.S. FDA if the study's aim is to investigate whether the supplement may be used to “diagnose, treat, cure, or prevent any disease.” This verbiage is from the FDA's definition of a drug, which is quite different from the legal definition of a dietary supplement. If, however, the study's goal is to simply characterize the pharmacodynamics (i.e., blood pressure measurement) or pharmacokinetics (i.e., rate and extent of oral absorption) of a phytochemical marker compound(s), then an IND is typically not required. To best determine whether an IND may be needed for a particular study involving a dietary supplement, investigators are encouraged to contact the FDA in advance.

## Conclusion

Clinical studies of dietary supplements based on botanical extracts are particularly challenging due to the complex nature of the extracts. Ensuring the design of the clinical study accurately tests the experimental question will depend on keeping the clinically relevant endpoints in mind when planning the pre-clinical studies and careful attention to the seemingly minor details that will ultimately determine the success of translating preclinical studies to the clinical setting.

## Data Availability Statement

The original contributions presented in the study are included in the article. Further inquires can be directed to the corresponding author/s.

## Author Contributions

ZF and BG wrote the manuscript. DR, IR, DH, and JR critically reviewed the article. All authors gave final approval for all aspects of the work, agreed to be fully accountable for ensuring the integrity and accuracy of the work, and read and approved the final manuscript.

## Funding

This work was supported by the National Center for Complementary and Integrative Health and the Office of Dietary Supplements of the NIH (P50 AT-002776 and R21 AT011107).

## Conflict of Interest

The authors declare that the research was conducted in the absence of any commercial or financial relationships that could be construed as a potential conflict of interest.

## Publisher's Note

All claims expressed in this article are solely those of the authors and do not necessarily represent those of their affiliated organizations, or those of the publisher, the editors and the reviewers. Any product that may be evaluated in this article, or claim that may be made by its manufacturer, is not guaranteed or endorsed by the publisher.
